# PEPT2 mRNA在肺纤维化大鼠肺组织中的表达

**DOI:** 10.3779/j.issn.1009-3419.2013.10.08

**Published:** 2013-10-20

**Authors:** 莉 李, 殿华 王, 旋 张, 鑫 宋, 晓骉 马, 早秀 胡

**Affiliations:** 1 650118 昆明，昆明医科大学第三附属医院云南省肿瘤医院生物治疗中心 Department of Bio-immunical Therapy, the Third Affiliated Hospital of Kunming Medical University, Kunming 650118, China; 2 650500 昆明，昆明医科大学云南省天然药物药理重点实验室 Yunnan Phamacological Laboratory of Natural Products, Kunming Medical University, Kuming 650500, China; 3 650118 昆明，昆明医科大学第三附属医院云南省肿瘤医院病理科 Department of Pathology, the Third Affiliated Hospital of Kunming Medical University, Kunming 650118, China

**Keywords:** 肽转运载体2, 博莱霉素, 肺纤维化, 羟脯氨酸, Peptide transporter 2, Bleomycin, Pulmonary fibrosis, Hydroxyproline

## Abstract

**背景与目的:**

肺纤维化是肺癌放化疗后的常见病理改变，是阻碍药物转运到肺部的关键因素之一，肽转运载体已经成为合理设计肽和肽类药物的靶标，本研究旨在探讨肽转运载体2（peptide transporter 2, PEPT2）mRNA在肺纤维化大鼠肺组织中的表达。

**方法:**

健康SD大鼠50只，随机分为5组。博莱霉素（bleomycin, BLM）7 d、14 d、28 d组：气管内一次性滴入博莱霉素溶液复制肺纤维化大鼠模型，分别于给药后7 d、14 d和28 d放血处死；生理盐水组滴入等量生理盐水，于14 d放血处死；正常组不做任何处理。各组取肺组织，光镜观察组织病理变化；检测样本羟脯氨酸含量；半定量RT-PCR检测肺组织PEPT2 mRNA表达。

**结果:**

BLM 7 d组大鼠肺组织呈急性炎症性改变，无纤维增生；BLM 14 d组和28 d组大鼠肺组织均有纤维化改变，以28 d组最为明显。BLM 7 d组肺组织羟脯氨酸含量与正常对照组和生理盐水组相比无统计学差异（*P* > 0.05）；14 d组和28 d组大鼠肺组织羟脯氨酸含量均高于正常对照组和生理盐水组（*P* < 0.05）。各组肺组织PEPT2 mRNA的相对表达量无统计学差异（*P* > 0.05）。

**结论:**

PEPT2 mRNA在博莱霉素致肺纤维化大鼠肺组织表达水平无明显变化，PEPT2可能是设计肺纤维化的新型肽类药物靶标之一。

## Abstract (en)

**Background and objective:**

Pulmonary fibrosis is a common pathological phenomenon in lung cancer patients after chemotherapy or radiotherapy. It is also a key hindrance to the transport of drugs to lung tissue. Peptide transporters have become a target of the rational design of peptides and peptide drugs. The aim of this study is to investigates the expression of peptide transporter 2 (PEPT2) mRNA in the lungs of rats with bleomycin (BLM)-induced pulmonary fibrosis.

**Methods:**

Fifty healthy adult Sprague-Dawley rats were randomly divided into five groups. One group was untreated (control), the second group was injected with normal saline solution (NS), and the three remaining groups were treated with a single dose of bleomycin to induce pulmonary fibrosis (BLM). Rats from the NS group were killed by exsanguination on day 14. Rats from the BLM group were killed by exsanguination on days 7, 14, and 28. The lung samples were observed under light microscopy and the hydroxyproline concentration was determined. The expression levels of PEPT2 mRNA were measured by RT-PCR.

**Results:**

The morphological study showed that collagenous fiber proliferated in the lungs of rats injected with BLM, indicating pulmonary fibrosis. This proliferation was apparent at 14 d post-injection and especially at 28 d post-injection. Hydroxyproline levels increased seven days post-injection compared with the control group and NS group, but there was no significant statistical difference (*P* > 0.05). Hydroxyproline levels significantly increased (*P* < 0.05) 14 d and 28 d post-infection. The change in the lung tissue pathology coincided with the change in hydroxyproline levels. There were no significant changes of pulmonary PEPT2 mRNA expression levels among the different groups (*P* > 0.05).

**Conclusion:**

PEPT2 is a potential peptide drug target in the treatment of pulmonary fibrosis, although there were no significant changes of PEPT2 mRNA expression in the lungs of rats with bleomycin-induced pulmonary fibrosis.

肺纤维化（pulmonary fibrosis, PF）是肺的炎症改变及纤维重塑同时存在的一种疾病。在肺癌的放化疗过程中，如博来霉素（bleomycin, BLM）的应用常常可以导致非特异性肺炎和肺纤维化^[1, 2]^。气道上皮细胞和肺泡上皮细胞中的药物浓度与肺纤维化疾病疗效密切相关，肺泡和支气管上皮就成了药物运输和治疗的主要靶标^[3]^。因此，如何将高浓度的药物转运到肺部是治疗肺纤维化疾病的关键。

肽转运载体2（peptide transporter 2, PEPT2）定位于支气管上皮细胞、Ⅱ型肺泡上皮细胞和小血管的内皮细胞中，可以通过底物转位与作为驱动力的跨膜电化学质子梯度相偶联，转运肽和肽类药物。转运效率较高，并可以降低给药剂量，减少全身副反应的发生^[4, 5]^。目前，肽转运载体已经成为合理设计肽和肽类药物如抗肿瘤、抗生素及抗病毒药物的靶标。因此，研究PEPT2在治疗肺疾病中的作用具有重要意义。

## 1 材料与方法

### 1.1 材料

健康SD大鼠50只（200 g±20 g），雌雄各半，由昆明医科大学实验动物中心提供；博莱霉素（日本化药株式会社）；羟脯氨酸检测试剂盒（南京建成生物工程研究所）；引物由上海生工生物工程技术服务有限公司合成。

### 1.2 方法

#### 1.2.1 实验分组与动物模型复制

50大鼠随机分为5组，每组10只。正常对照组不作任何处理；生理盐水组：气管内一次性滴入0.2 mL生理盐水，14 d后予股动脉放血处死；博莱霉素7 d、14 d、28 d组：气管内一次性滴入0.5%的BLM溶液5 mg·kg^-1^，复制肺纤维化模型，分别于给药后7 d、14 d和28 d予股动脉放血处死取肺组织。

#### 1.2.2 肺组织形态学观察

股动脉放血处死实验动物，开胸取右肺下叶，用10%中性甲醛固定24 h后，常规石蜡包埋、切片，HE染色和GS染色后光镜下观察肺组织形态结构。

#### 1.2.3 肺组织中羟脯氨酸（hydroxyproline, HYP）含量测定

采用碱水解法测定大鼠肺组织中HYP的含量，反映肺纤维化的程度。

#### 1.2.4 RT-PCR检测

取肺组织样品100 mg，将组织剪碎，加入1 mL Trizol。用匀浆器充分匀浆，用氯仿、异丙醇、75%乙醇提取肺组织总RNA。采用第一条链合成试剂盒合成cDNA，PEPT2序列5’-GCTGCCTACTGAAGCCAAATGCTTG-3’及5’-AGAGGCTGCTGAAGGCATGGT-3’；内参照采用GAPDH，引物序列为5’-ACAGCAACAGGGTGGTGGAC-3’及5’-TTTGAGGGTACAGCGAACTT-3’，扩增片段长度为327 bp。PCR反应：模板浓度为60 pg，94 ℃变性1 min，55 ℃退火1 min，72 ℃延伸1 min，扩增28个循环，72 ℃延伸10 min。

#### 1.2.5 RT-PCR产物半定量测定

由上海生工生物有限公司进行PCR产物测序，使用FASTA与基因库中大鼠PEPT2序列进行同源性比较；用GelDoc凝胶成像系统进行检测，并用Quantity One软件对各阳性条带的光密度进行测定，以PEPT2与GAPDH的光密度比值表示PEPT2 mRNA的相对表达量。

### 1.3 统计学处理

采用SPSS 17.0进行统计分析，组间比较采用单因素方差分析和SNK-*q*检验，结果用Mean±SD表示，以*P* < 0.05为差异具有统计学意义。

## 2 结果

### 2.1 BLM致大鼠肺纤维化组织形态学变化

HE染色光镜观察发现：BLM 7 d组大鼠肺泡腔内渗出物增多，局部有少量出血，肺泡壁内和肺间质内有中性粒细胞、淋巴细胞浸润，可见少量成纤维细胞，存在局部萎陷及小血管壁增厚，呈弥漫性肺泡炎改变；BLM 14 d组大鼠部分肺组织呈片状实变，肺泡上皮细胞增生，肺泡壁增宽，片状肺泡腔缩小或消失，个别肺泡腔内可见泡沫细胞，肺泡间质内可见少量炎细胞浸润，细支气管上皮细胞增生，腔内可见脱落坏死的上皮细胞；BLM 28 d组大鼠部分肺组织呈片状实变，肺泡腔广泛的缩小或消失，肺泡壁增宽，纤维化；在肺泡间质、肺泡腔内可见较多淋巴细胞、浆细胞等炎性细胞浸润，肺泡壁毛细血管扩张，见图 1。

**1:**
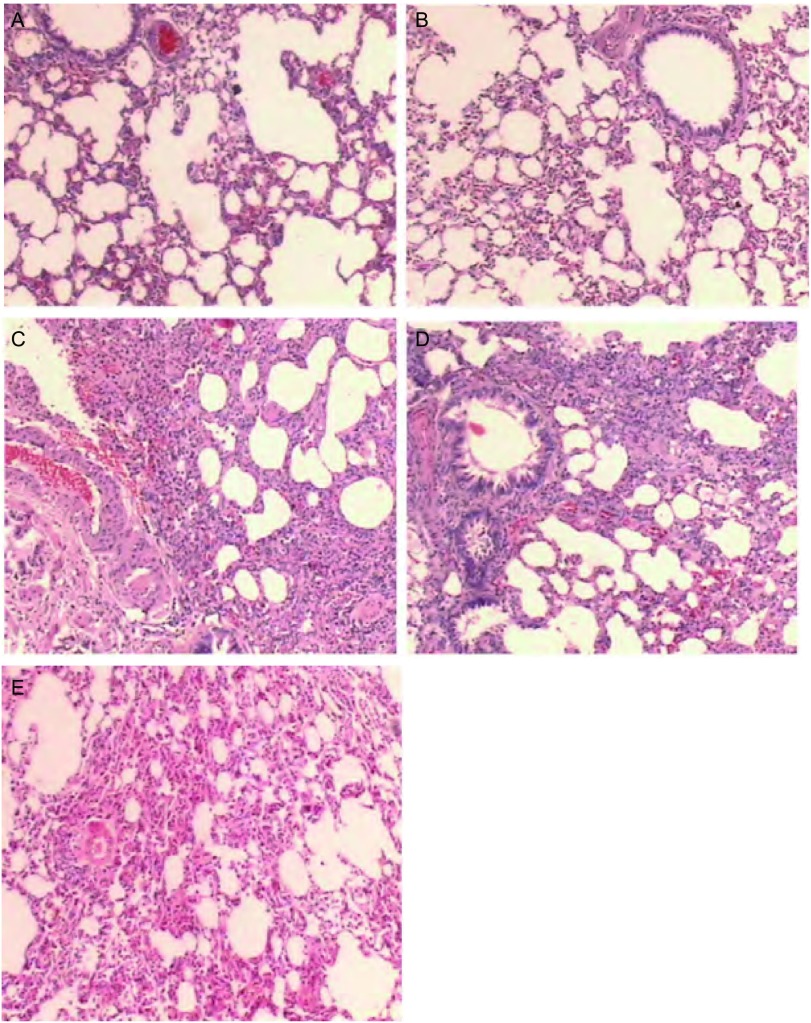
各组大鼠肺组织的病理改变（HE，×80）。A：对照组；B：生理盐水组；C：博来霉素7天组；D：博来霉素14天组；E：博来霉素28天组。 Histopathologic changes of lung tissue in each group (HE, ×80). A: control group; B: normal saline solution (NS) group; C: bleomycin (BLM) 7 d group; D: BLM 14 d group; E: BLM 28 d group.

GS染色结果显示：BLM 14 d组大鼠肺内胶原纤维数量及分布基本正常，局部网状纤维大量增生；BLM 28d组大鼠肺内大量增生的网状纤维中出现增生的胶原纤维岛，见图 2。

**2:**
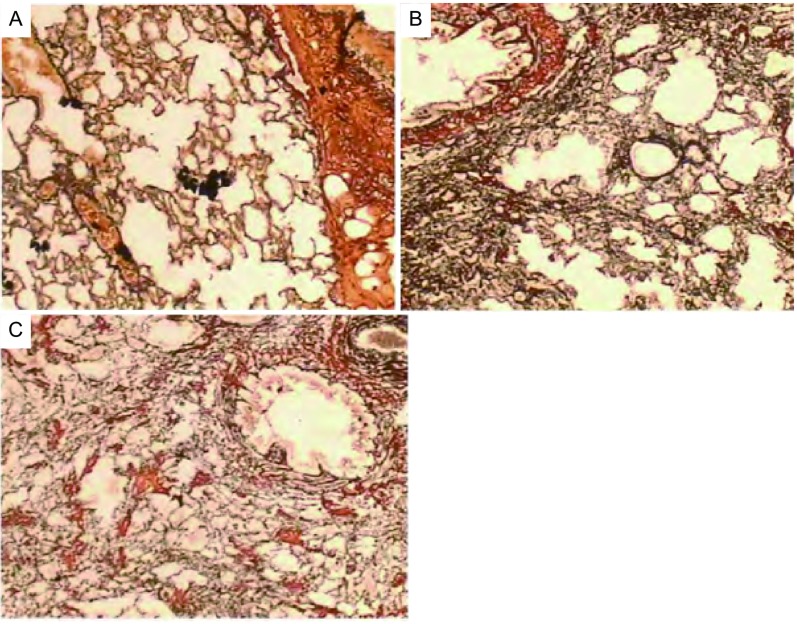
生理盐水组（A）、BLM 14 d（B）和28 d（C）组大鼠肺组织GS染色图（×80） GS staining of lung tissue of NS (A), BLM 14 d (B) and 28 d (C) groups (×80)

### 2.2 BLM致肺纤维化大鼠肺组织羟脯氨酸含量的变化

BLM致大鼠肺纤维化过程中，大鼠肺组织HYP含量随时间增长逐渐升高，BLM 7 d组肺组织HYP含量与正常对照组、生理盐水组相比无统计学差异（*P* > 0.05），14 d组和28 d组大鼠肺组织HYP含量依次增高，且均高于正常对照组和生理盐水组（*P* < 0.05），见图 3。

**3:**
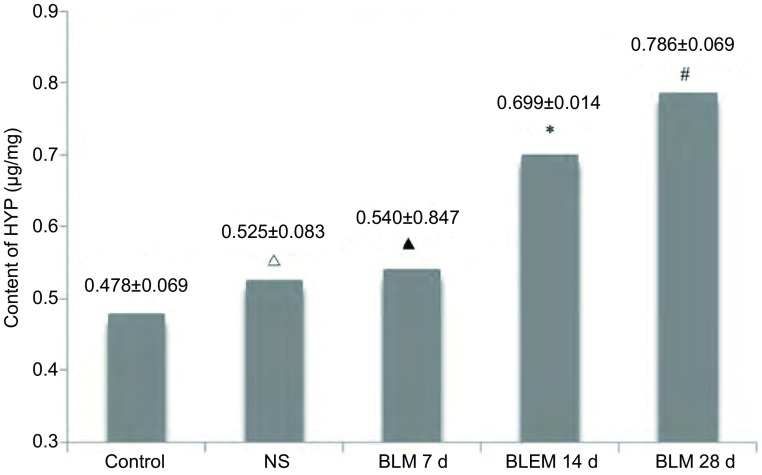
各组大鼠肺组织的HYP含量 Content of hydroxyproline (HYP) of lung tissue in each group. *Vs* control group: ^△^*P*=0.240; ^▲^*P*=0.132; ^*^P < 0.001; ^#^*P* < 0.001.

### 2.3 半定量

RT-PCR检测肺纤维化大鼠肺组织PEPT2 mRNA表达RT-PCR产物测序结果与GenBank中大鼠PEPT2序列进行同源性比较，结果显示与GenBank中大鼠PEPT2（基因登录号：D63149，gi: 1374711）cDNA序列几乎完全一致，同源性高达99.3%（284/286），RT-PCR产物序列（1 bp-284 bp）与大鼠PEPT2 cDNA序列的154 bp-437 bp完全一致。此结果证实了RT-PCR产物为大鼠PEPT2 cDNA片段。

各组大鼠肺组织PEPT2和GAPDH凝胶电泳图，用凝胶成像系统进行检测可见PEPT2的长度为327 bp，内参GAPDH的长度为252 bp，见图 4A。测定PEPT2 mRNA和GAPDH阳性条带的光密度百分数，以PEPT2与内参GAPDH的光密度百分数的比值表示相对表达量，各实验组肺组织PEPT2 mRNA相对表达量的差异无统计学意义（*P* > 0.05），见图 4B。

**4:**
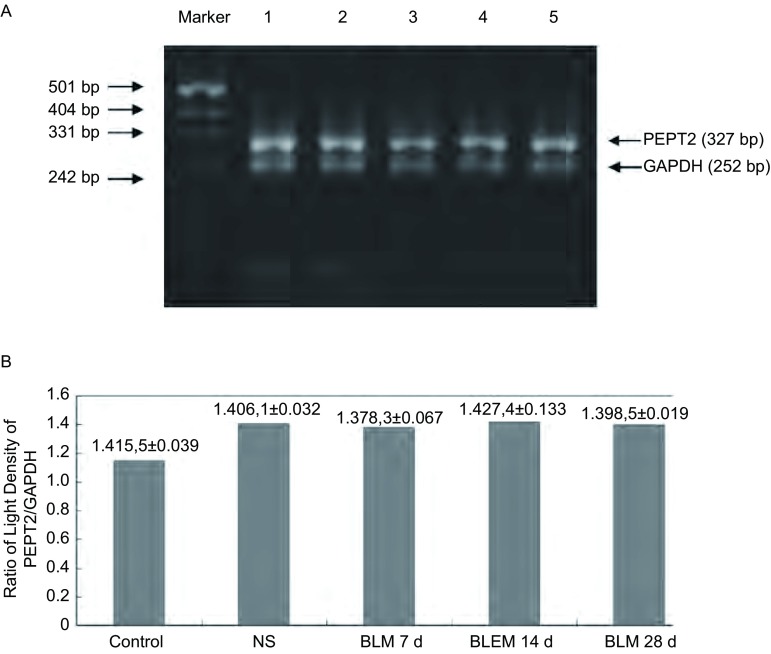
各组大鼠肺组织中PEPT2 mRNA和GAPDH mRNA的变化。A：各组大鼠肺组织中PEPT2 mRNA和GAPDH mRNA的凝胶电泳图。1：对照组；2：NC组；3：BLM 7 d组；4：BLM 14 d组；5：BLM 28 d组；B：柱状图显示各组大鼠肺组织中PEPT2 mRNA和GAPDH mRNA光密度比值无统计学差异（*P*=0.696）。 The change of PEPT2 mRNA and GAPDH mRNA of lung tissue in each group. A: Agarose gel electrophoresis of PEPT2 mRNA and GAPDH mRNA of lung tissue in each group.1: control group; 2: NS group; 3: BLM 7 d group; 4: BLM 14 d group; 5: BLM 28 d group; B: Compared the ratio of light density of PEPT2 and GAPDH mRNA which were products of RT-PCR each group in the lung tissue of rat. *Vs* betweem groups: *P*=0.696.

## 3 讨论

PEPT2属于H^+^依赖性寡肽转运载体家族（family of proton-dependent oligopeptide transporter, POT），可在肺、肾脏、大脑、脾和乳腺等组织表达^[6, 7]^。在肺部，PEPT2 mRNA主要在支气管上皮细胞、肺泡Ⅱ型上皮细胞和小血管的内皮细胞表达^[4, 5]^。PEPT2独特的分子特征及其组织分布特点，可以将高浓度的抗生素、抗病毒药物运输到气道上皮细胞和肺泡上皮细胞中^[4, 5]^。据报道^[4-7]^PEPT2可转运仿肽类药物如β-内酰胺抗生素、苯丁亮氨酸、肾素抑制剂。因此，肺组织中PEPT2 mRNA的表达量与药物转运效率密切相关。

放射治疗是中晚期肺癌综合治疗的重要治疗手段之一，且常常引起放射性肺损伤，最终导致肺纤维化^[1, 2]^。广泛应用的化疗药物如博来霉素，使用本药10%-23%患者可发生肺毒性，表现为呼吸困难、咳嗽、胸痛、肺部啰音等，导致非特异性肺炎和肺纤维化，甚至快速死于肺纤维化^[2, 8, 9]^。肺纤维化是一种高致死率疾病，至今尚无有效的治疗策略能阻止其自然进程和致死性^[3]^。研究^[4, 5]^表明，气道上皮细胞和肺泡上皮细胞中的药物浓度与肺纤维化疾病疗效密切相关。因此，如何将高浓度的药物转运到肺部是治疗肺纤维化疾病的关键。研究PEPT2蛋白在肺纤维化大鼠肺组织的表达，可有效地将治疗肺纤维化疾病的新型肽类药物及前体药物运输到肺部，提高肺组织局部的药物浓度，从而促进治疗肺纤维化疾病的新型药物和新的治疗策略的发展。

1996年，德国Boll教授^[10]^首先在肾脏发现具有高亲和力的肽及肽类药物转运蛋白PEPT2，并鉴定其分子结构。1999年Grondberg^[11]^首次通过Northerning blot和RT-PCR技术研究表明PEPT2可在正常大鼠呼吸道中表达。随后开展了针对正常动物肺组织中PEPT2的结构、功能和分子特征的大量研究^[4, 6, 7, 12, 13]^。但迄今为止，国内外对肺部疾病状态下PEPT2 mRNA在动物肺部表达水平的研究较少。

动物模型是否复制成功有一定的评价指标，目前评价该模型是否建立，最主要的指标是对肺组织进行病理组织学评价，其次还有肺胶原蛋白及纤维蛋白的代谢产物含量的测定等^[9]^。本研究在成功复制的肺纤维化大鼠动物模型上，采用半定量RT-PCR技术检测PEPT2 mRNA在大鼠肺部的表达水平。结果发现BLM各组大鼠肺组织PEPT2 mRNA表达水平与正常组大鼠和生理盐水组大鼠相比无统计学差异（*P* > 0.05）。这表明PEPT2 mRNA在BLM致肺纤维化大鼠肺组织表达水平无明显变化。

PEPT2为高亲和力低容量转运载体，正常状态下可转运大量药理活性底物致肺部，肺部疾病状态下，由于PEPT2 mRNA在肺纤维化大鼠肺组织表达水平无明显变化，表明在肺纤维化时，可以通过肺部给药方式给予肽和肽类药物，降低药物的蛋白分解作用和避免肝脏的首过效应，使其通过PEPT2蛋白转运作用，将高浓度的药物转运到靶位，提高局部的药物浓度，增加其生物利用度，从而提高药物疗效。本实验通过研究药物作用的靶点——肽转运载体PEPT2蛋白在肺纤维化状态下的表达情况，可为研制高效低毒的新型肽类药物提供实验依据。
